# Neurobiologically Realistic Determinants of Self-Organized Criticality in Networks of Spiking Neurons

**DOI:** 10.1371/journal.pcbi.1002038

**Published:** 2011-06-02

**Authors:** Mikail Rubinov, Olaf Sporns, Jean-Philippe Thivierge, Michael Breakspear

**Affiliations:** 1 Black Dog Institute and School of Psychiatry, University of New South Wales, Sydney, Australia; 2 Mental Health Research Division, Queensland Institute of Medical Research, Brisbane, Australia; 3 CSIRO Information and Communication Technologies Centre, Sydney, Australia; 4 Department of Psychological and Brain Sciences, Indiana University, Bloomington, Indiana, United States of America; University of California Santa Barbara, United States of America

## Abstract

Self-organized criticality refers to the spontaneous emergence of self-similar dynamics in complex systems poised between order and randomness. The presence of self-organized critical dynamics in the brain is theoretically appealing and is supported by recent neurophysiological studies. Despite this, the neurobiological determinants of these dynamics have not been previously sought. Here, we systematically examined the influence of such determinants in hierarchically modular networks of leaky integrate-and-fire neurons with spike-timing-dependent synaptic plasticity and axonal conduction delays. We characterized emergent dynamics in our networks by distributions of active neuronal ensemble modules (neuronal avalanches) and rigorously assessed these distributions for power-law scaling. We found that spike-timing-dependent synaptic plasticity enabled a rapid phase transition from random subcritical dynamics to ordered supercritical dynamics. Importantly, modular connectivity and low wiring cost broadened this transition, and enabled a regime indicative of self-organized criticality. The regime only occurred when modular connectivity, low wiring cost and synaptic plasticity were simultaneously present, and the regime was most evident when between-module connection density scaled as a power-law. The regime was robust to variations in other neurobiologically relevant parameters and favored systems with low external drive and strong internal interactions. Increases in system size and connectivity facilitated internal interactions, permitting reductions in external drive and facilitating convergence of postsynaptic-response magnitude and synaptic-plasticity learning rate parameter values towards neurobiologically realistic levels. We hence infer a novel association between self-organized critical neuronal dynamics and several neurobiologically realistic features of structural connectivity. The central role of these features in our model may reflect their importance for neuronal information processing.

## Author Summary

The intricate relationship between structural brain connectivity and functional brain activity is an important and intriguing research area. Brain structure (the pattern of neuroanatomical connections) is thought to strongly influence and constrain brain function (the pattern of neuronal activations). Concurrently, brain function is thought to gradually reshape brain structure, through processes such as activity-dependent plasticity (the “what fires together, wires together” principle). In this study, we examined the relationship between brain structure and function in a biologically realistic mathematical model. More specifically, we considered the relationship between realistic features of brain structure, such as self-similar organization of specialized brain regions at multiple spatial scales (hierarchical modularity) and realistic features of brain activity, such as self-similar complex dynamics poised between order and randomness (self-organized criticality). We found a direct association between these structural and functional features in our model. This association only occurred in the presence of activity-dependent plasticity, and may reflect the importance of the corresponding structural and functional features in neuronal information processing.

## Introduction

Self-organized criticality is increasingly postulated to underlie the organization of brain activity [1]–[2]. The notion of self-organized criticality describes an unsupervised emergence of critical dynamics in complex systems dominated by internal interactions [3]–[4]. Critical dynamics emerge at the transition between randomness (subcritical dynamics) and order (supercritical dynamics), and are characterized by self-similar (power-law-distributed) spatial and temporal properties of system events (e.g. neural activations). The occurrence of these dynamics in the brain is theoretically appealing and is increasingly empirically supported. Theoretically, and increasingly empirically, critical dynamics are associated with optimized information transmission and storage [5]–[8], maximized dynamic range [9]–[10] and successful learning [11]. Empirically, multielectrode array recordings of spontaneous activity from organotypic cortical slice cultures [5]–[6] and dissociated cortical neuron cultures [12]–[13] show power-law scaling of distributed “avalanche” activity of neuronal ensembles. Multielectrode array recordings of spontaneous cortical activity in the awake rhesus monkey also show power-law scaling of avalanches [14], suggesting that these dynamics are not confined to *in vitro* preparations. The temporal and spatial statistics of EEG, ECoG, MEG and fMRI signals likewise show power-law scaling [15]–[18], although the relationship of these large-scale brain signals to avalanches of neuronal ensembles may not be straightforward.

Brain dynamics are thought to be strongly influenced by neuroanatomical connectivity [19]–[22]. Consequently, self-organized critical brain dynamics may be influenced by properties of neuroanatomical organization, such as hierarchical modularity, small-worldness and economical wiring [23]–[26]. Hierarchical modularity is a self-similar organization in which functionally specialized neural clusters (e.g. cortical lobes) contain smaller and more specialized neural clusters (e.g. cortical nuclei, cortical columns) at multiple spatial scales. Small-worldness is an organization which combines modularity and robust between-module connectivity. Economical wiring is an organization which contains predominantly short connections.

The presence of an intuitive association between self-similar brain structure (i.e. hierarchical modularity) and self-similar brain dynamics (i.e. self-organized criticality), has not been previously examined. The relationship between brain structure and dynamics is reciprocal: while the structure strongly constrains the dynamics, the dynamics continuously modify the structure through mechanisms such as activity-dependent synaptic depression [27] and spike-timing-dependent plasticity [28]–[29]. We previously showed that this reciprocal relationship is associated with an unsupervised emergence of modular small-world structural connectivity, in a large-scale model of spontaneous brain activity [30]. We now ask whether realistic structural organization is associated with the emergence of self-organized critical dynamics.

A number of modeling studies recently reported self-organized critical avalanche dynamics in neuronal networks with nontrivial topology and activity-dependent plasticity [31]–[33]. These studies focused on conceptual features of network organization and plasticity, and hence omitted neurobiologically realistic features such as membrane leakage, axonal delays and spike-timing-dependent plasticity. Other studies are increasingly beginning to examine these relationships in more realistic networks [34]–[35]. Most studies however, remain constrained by assessment of power-law distributions with unreliable linear least-squares-based methods [36]. In contrast, we aim to systematically and rigorously examine the relationship between anatomical connectivity, synaptic plasticity and self-organized criticality, in a realistic network model of neuronal activity. To this end, we extend a recent model of nonperiodic synchronization in networks of leaky integrate-and-fire neurons [37] to incorporate large, sparse, hierarchical modular connectivity, spike-timing-dependent plasticity and other neurobiologically realistic features such as axonal conduction delays and neuronal inhibition. We hypothesize that the neurobiologically realistic features of our model will facilitate the emergence of self-organized critical dynamics.

## Methods

**Figure 1:**
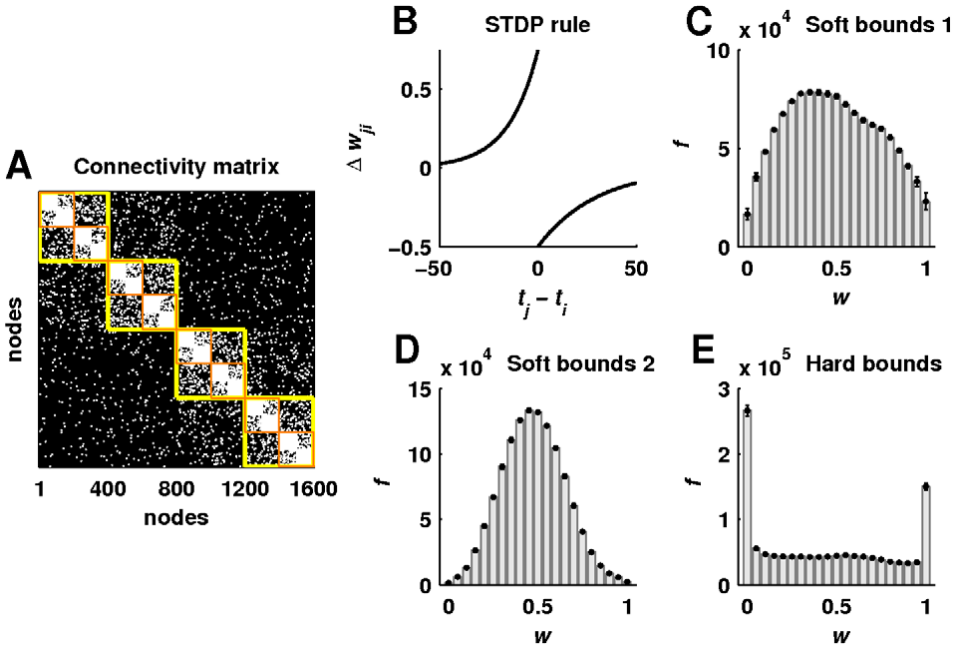
Hierarchically modular connectivity and spike-timing-dependent plasticity. (a) An illustrative connectivity matrix of a hierarchical modular network. This network consists of sixteen 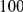-neuron modules, organized into four hierarchical levels. Squares in the connectivity matrix outline the nesting of hierarchical level 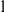 (small orange squares) inside hierarchical level 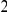 (large yellow squares). In the present study we considered networks of 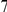 hierarchical levels and 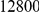 neurons. (b) An illustration of the synaptic plasticity rule used in the study. (c) Weight frequency distributions for the STDP rule with soft bounds (used in most simulations). (d) Weight frequency distributions for the STDP rule with soft bounds and reduced learning rate (used in some simulations). (e) Weight frequency distributions for the STDP rule with hard bounds (used in some simulations). Error bars represent the standard deviation.

The fundamental organizational unit of our network model is a densely connected 100-neuron module [37]. All networks comprised 128 of these modules, and the modules were organized into seven hierarchical levels (Figure 1a). The number and location of intermodule connections in these levels was determined by specific (power-law, exponential or linear) scaling functions. Neuronal subthreshold membrane dynamics were integrated exactly and neuronal spike times were interpolated between fixed time steps. Network dynamics were characterized by probability distributions of module avalanche sizes and durations. For each configuration, the presence of power-law scaling in these distributions was rigorously assessed. All computations were performed in Matlab using custom-written compiled C-language code (provided as supplementary information to this article, Text S1, S2, S3, S4, S5, S6, S7, S8, S9, S10, S11, S12, S13, S14, S15, S16, and S17).

### Spiking neuron model with synaptic plasticity

The studied leaky integrate-and-fire neuron evolves according to
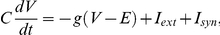
where 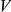 is the membrane potential, 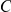 is the membrane capacitance, 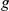 is the leakage conductance, 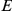 is the resting potential and 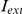 and 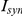 are the external current and synaptic current, respectively. When 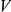 exceeds a constant threshold 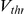, the neuron is said to spike and 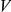 is reset to the value 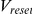 for an absolute refractory period 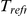. The external current maintains a constant low level of background neuronal activity, while synaptic currents couple anatomically connected neurons. In the model, we set 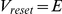 and 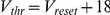. We set 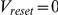 for clarity, but any other value (e.g. 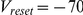) results in equivalent dynamics, as long as the above relationship between 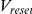, 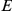 and 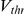 holds. We discuss these and other aspects of the integration scheme in the Supplementary Information (Text S1).

For a postsynaptic neuron 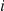, we modeled synaptic currents with decaying exponentials,
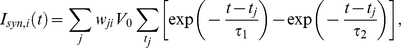
where the outer sum is over all presynaptic neighbors of 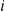, the inner sum is over all previous spike times 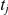 of each presynaptic neighbor 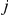, 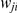 is the synaptic weight from 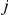 to 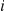, 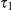 and 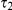 are the slow and fast decay constants, and 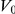 is a magnitude parameter. Synaptic coupling incorporated axonal delays, set to uniformly distributed random integers between 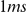 and 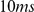. These values are in the range of empirically estimated axonal delays [38]. For computational simplicity we used the same distribution of axonal delays for all hierarchical levels. We note that long-range cortical connections are often more thickly myelinated than short-range connections so there is no simple relationship between inter-level distance and axonal delay.

Synaptic weights changed at every spike of a neuron incident to the synapse, according to a spike-timing-dependent plasticity (STDP) rule (Figure 1b). The STDP rule potentiates 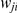 when the postsynaptic neuron 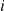 spikes shortly after the presynaptic neuron 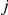, and depresses 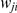 when neuron 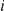 spikes shortly before neuron 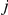. More specifically, when 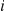 or 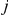 spike, 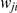 changes as 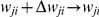, with
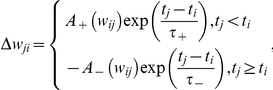
where 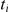 and 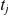 are the latest spike times of 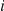 and 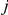, 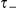 and 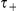 are time constants and 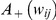 and 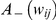 are weight dependence functions,
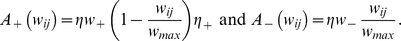
The weight dependence functions keep all weights between 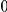 and 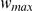, and rescale weight changes by the weight constants 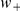 and 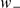, and by the rate constant 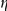. The above functions enable soft weight bounds, or multiplicative weight dependence. Alternative functions,
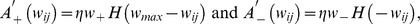
where 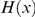 is the Heaveside step function, enable hard weight bounds, or additive weight dependence. The choice between soft and hard weight bounds has important implications for synaptic weight distributions (Figure 1c–e). The unimodal distribution associated with soft weight bounds has more experimental support [39], although both hard and soft weight bounds are extensively used in computational studies. We used soft bounds in most simulations, but also explored the robustness of our results to the presence of hard bounds.

Parameter values of the model were adapted from the Thivierge and Cisek [37] study and are shown in Table 1. In the present study, we find that the postsynaptic-response magnitude and STDP learning rate parameters facilitate important internal interactions in the network. We show that high values of these parameters are required to compensate for the relatively small number of neuronal synapses in our networks. We also show that these values may be substantially reduced in larger networks with greater numbers of synapses.

**Table 1:** Default parameter values of the spiking neuron model.

Integration parameters	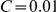 , 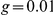 , 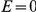 , 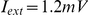
Neuronal spike parameters	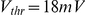 , 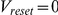 , 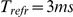
Post synaptic response parameters	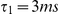 , 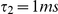 , 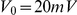
STDP parameters	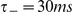 , 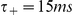 , 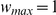 , 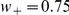 , 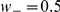 , 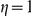

### Hierarchical modular connectivity

**Figure 2:**
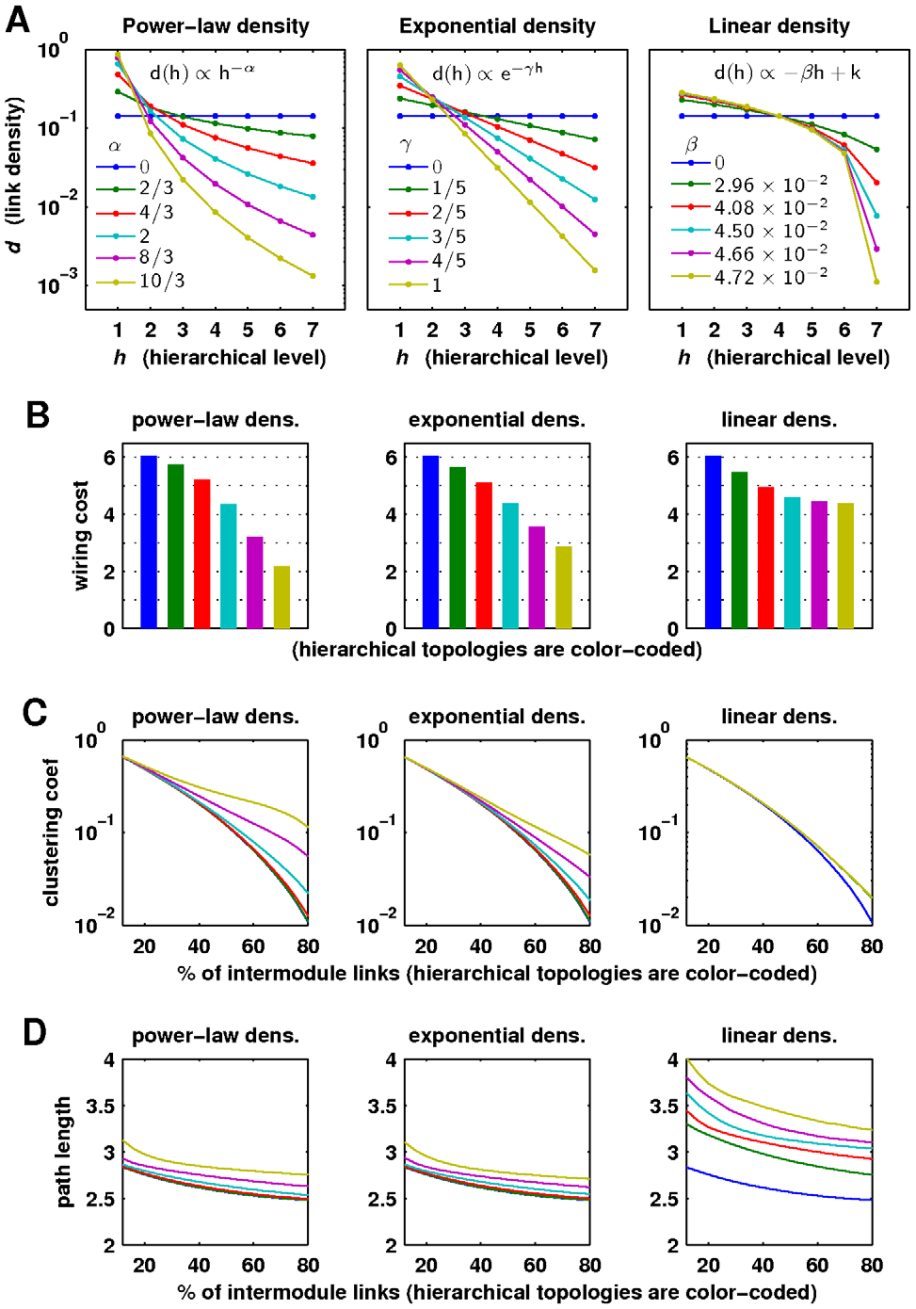
Properties of hierarchically modular connectivity. (a) Power-law, exponential and linear density scaling functions of the networks used in this study. (b) Dimensionless measures of wiring cost for each density scaling function in (a). The wiring cost was computed by equating synaptic cost with hierarchical-level number, and averaging the cost over all synapses. Hence, synapses in hierarchical level 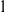 were assigned a cost of 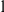, synapses in hierarchical level 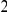 were assigned a cost of 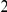, etc. Approximate values of the network (c) clustering coefficient and (d) characteristic path length across a range of randomizations of each hierarchical topology. Color-coding is the same as in (a).

Each network comprised 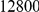 neurons, subdivided into 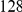 modules. Each module comprised 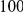 neurons, of which 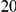 neurons were inhibitory and 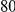 excitatory. Inhibitory neurons only formed synaptic connections with all 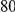 excitatory within-module neurons. On the other hand, excitatory neurons could potentially form synaptic connections with excitatory or inhibitory neurons in all modules. Initially, excitatory neurons only formed synaptic connections with all 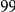 other within-module neurons. Subsequently, excitatory synapses were probabilistically rewired within seven hierarchical levels (Figure 1a). The density of intermodular connections, 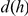, within each level 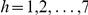, was set using power-law (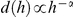), exponential (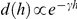) or linear (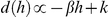) scaling functions, with 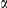, 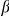 and 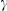 determining density drop-off rates (Figure 2a). Synapses were rewired in a way that preserved the total number of synapses per neuron [40] but not connection reciprocity. For each network, rewiring occurred progressively from the outermost to the innermost hierarchical level. The location of synapses in each network was kept fixed during simulations.

The wiring cost associated with each scaling function was computed by estimating the number of synapses in each hierarchical level for that function, equating the cost of each synapse with the number of its hierarchical level (e.g. synapses in level 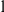 were assigned a cost of 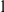), and averaging the cost over all synapses. Higher density drop-off rates were associated with lower wiring cost (Figure 2b). The low wiring cost was in turn associated with higher clustering coefficients and higher characteristic path lengths in the network (Figure 2b–d). Clustering coefficients and characteristic path lengths are simple measures of modular organization and between-module connectivity, respectively [41].

### Network dynamics and module spikes

We integrated subthreshold neuronal dynamics exactly, interpolated neuronal spike times between 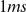 intervals and recorded neuronal activity at 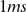 bins [42]. We began all simulations by setting all synaptic weights to 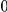 and setting all membrane potentials to uniformly distributed random values from 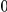 to 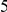. We discarded five minutes of initial activity, ensuring in each case that synaptic weights converged to a stable distribution. We recorded five minutes of subsequent activity and described this activity in terms of module spikes. Module spikes represent simultaneous activations of large numbers of within-module neurons, and hence correspond to network spikes described in empirical data [43]–[44]; we used the term module spike, rather than network spike, to avoid potential confusion with global network synchrony. We explicitly note that module spikes are conceptually distinct from individual neuron spikes. We determined the occurrence of module spikes with a shuffling algorithm that preserved individual spike frequency but destroyed global patterns of network activity. In this algorithm, spike times of all excitatory within-module neurons are randomly shuffled between active time bins. Module spikes are then said to occur when the number of simultaneously active neurons in the original data exceeds a threshold corresponding to the number of simultaneously active neurons in 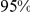 of the shuffled data. For each module, we averaged the spike threshold from 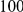 shuffled matrices.

**Figure 3:**
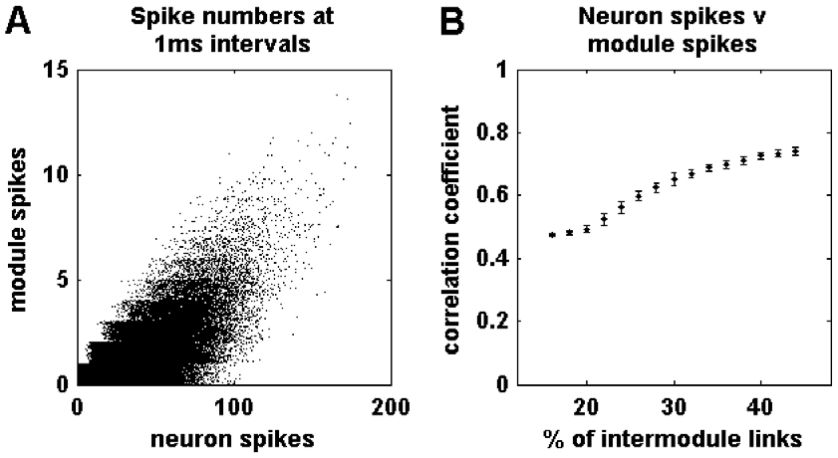
Relationship between neuron spikes and module spikes. (a) An illustrative scatter plot of the numbers of neuron spikes and module spikes at sampled 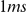 intervals. Integer spike numbers were jittered by the addition of uniformly distributed random numbers between 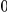 and 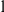. (b) Correlation coefficients between numbers of neuron spikes and numbers of module spikes as a function of network randomization. Correlations were computed from five-minute spike rasters. Error bars represent the standard error of the mean from 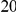 simulations.

It is also possible to describe network activity in terms of individual neuron spikes, rather than in terms of module spikes. In our simulations, neurons were likely to spike in module-specific groups, and neuronal spikes were hence strongly correlated with module spikes (Figure 3). We concentrated on module spike patterns because these describe activations of neuronal ensembles and have clear parallels with population spikes observed through changes of local field potentials in empirical studies of self-organized criticality [5], [12]. Neuronal spike patterns are studied in more detail elsewhere, e.g. in memory consolidation [45]. We also note that neuronal activity is likely to occur at every time point in large networks; consequently descriptions of avalanches of individual neuron spikes require a global network threshold to remove background activity. In our simulations, this threshold resulted in minimal event sizes of 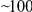 neurons, which, together with maximal event sizes of 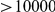 neurons, made rigorous detection of power-law scaling computationally prohibitive.

### Avalanche distributions and assessment of power-law scaling

We defined an avalanche as a sequence of temporally continuous (in 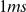 bins) module spikes, preceded and followed by a period of inactivity [5]. Correspondingly, we defined the avalanche size as the number of module spikes in the avalanche, and the avalanche duration as the total time between onset and conclusion of the avalanche. The minimal avalanche has size 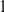 module and duration 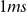. The maximal avalanche may be arbitrarily large because modules can be potentially active multiple times in the same avalanche. More realistically, the overwhelming majority of avalanches in our simulations, especially in simulations with neurobiologically realistic connectivities (Figure 7a), did not exceed the system size of 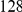 modules.

Probability distributions of avalanche sizes and durations allow a concise quantification of network dynamics. For instance, subcritical dynamics are characterized by small avalanche sizes and rapidly decaying avalanche size distributions, while supercritical dynamics are characterized by large avalanche sizes and slowly decaying avalanche size distributions. Critical dynamics are characterized by avalanche sizes and durations that follow power-law distributions,
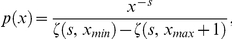
with a cumulative distribution function,
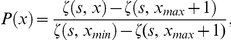
where 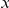 is avalanche size or duration, 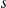 is the scaling exponent, 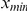 and 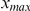 are upper and lower cut-offs and 
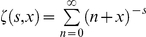
 is the generalized Hurwitz zeta function. The functions explicitly incorporate an upper cut-off 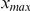, as distributions are necessarily bounded by system size [46]. In the following, we set 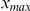 to the maximal event size in each distribution.

We rigorously assessed the presence of power-law scaling in avalanche distributions, by adapting the methods described in Clauset et al. [36]. We hence estimated 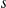 using the method of maximum likelihood. This method is mathematically robust and accurate for large number of samples 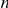 (in our simulations 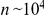), unlike linear least-squares-based methods commonly used in previous studies. For a given 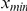, we estimated 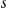 by numerically maximizing the log-likelihood function,
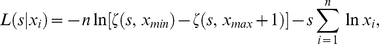
where 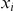, 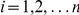 are the observed values of 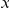, such that 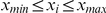 for all 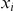. We imposed the condition 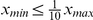 and this conservative condition ensured that we considered a wide range of events. We then chose the 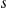, 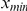 pair that minimized the Kolmogorov-Smirnov statistic,
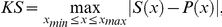
where 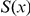 is the cumulative distribution function of the data and 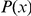 is the cumulative distribution function of the fitted model.

We formally assessed the power-law goodness-of-fit, by generating 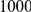 synthetic power-law distributions with equivalent 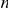, 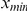, 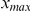 and 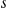. For each generated dataset we individually estimated 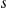 and 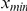, and computed the 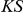 statistic as above. This procedure gives a 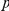-value as the fraction of instances in which the 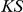 statistic of the generated data exceeds the 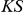 statistic of the original data. We deemed that 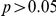 [47] did not allow to reject the power-law hypothesis, and hence suggested power-law scaling. Smaller or larger 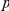-values (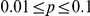) did not qualitatively change our results.

We imposed three additional conditions to ensure meaningful power-law scaling. Firstly, we required that maximal avalanche sizes approach system limits (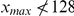 modules), to ensure that power laws did not reflect rapidly decaying subcritical dynamics. Secondly, we required that avalanche distributions extracted from corresponding shuffled module spike matrices had goodness-of-fit 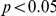. Thirdly, we directly compared power-law and exponential distribution fits, by computing the log-likelihood ratio for the best-fitting power-law and exponential distributions. The corresponding probability distribution, cumulative distribution and log-likelihood functions for the exponential distribution are,
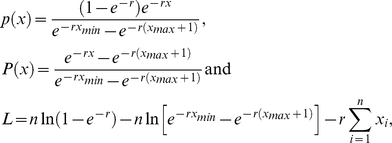
respectively, where 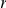 is the exponential parameter. The log-likelihood ratio compares two distributions and identifies a distribution which fits the data better. A significance test on the log-likelihood ratio gives a 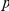-value on the statistical significance of this comparison [48], [36]]. We deemed that 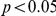 indicated a statistically significant difference in fit between distributions. We did not attempt to compare power-law and log-normal distribution fits because it is very difficult to differentiate these two distributions and hence such comparisons are typically inconclusive [36].

We summarized the presence of power-law scaling in each distribution with a single statistic 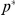. For each distribution, 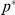 equaled the goodness-of-fit 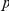-value for the power-law model if the distribution additionally fulfilled the above three conditions; alternatively 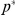 was set to 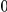. We averaged 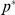 over 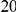 independent simulations for each type of connectivity, and considered 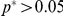 to indicate power-law scaling.

## Results

### Synaptic plasticity enabled a phase transition from subcritical to supercritical dynamics

**Figure 4:**
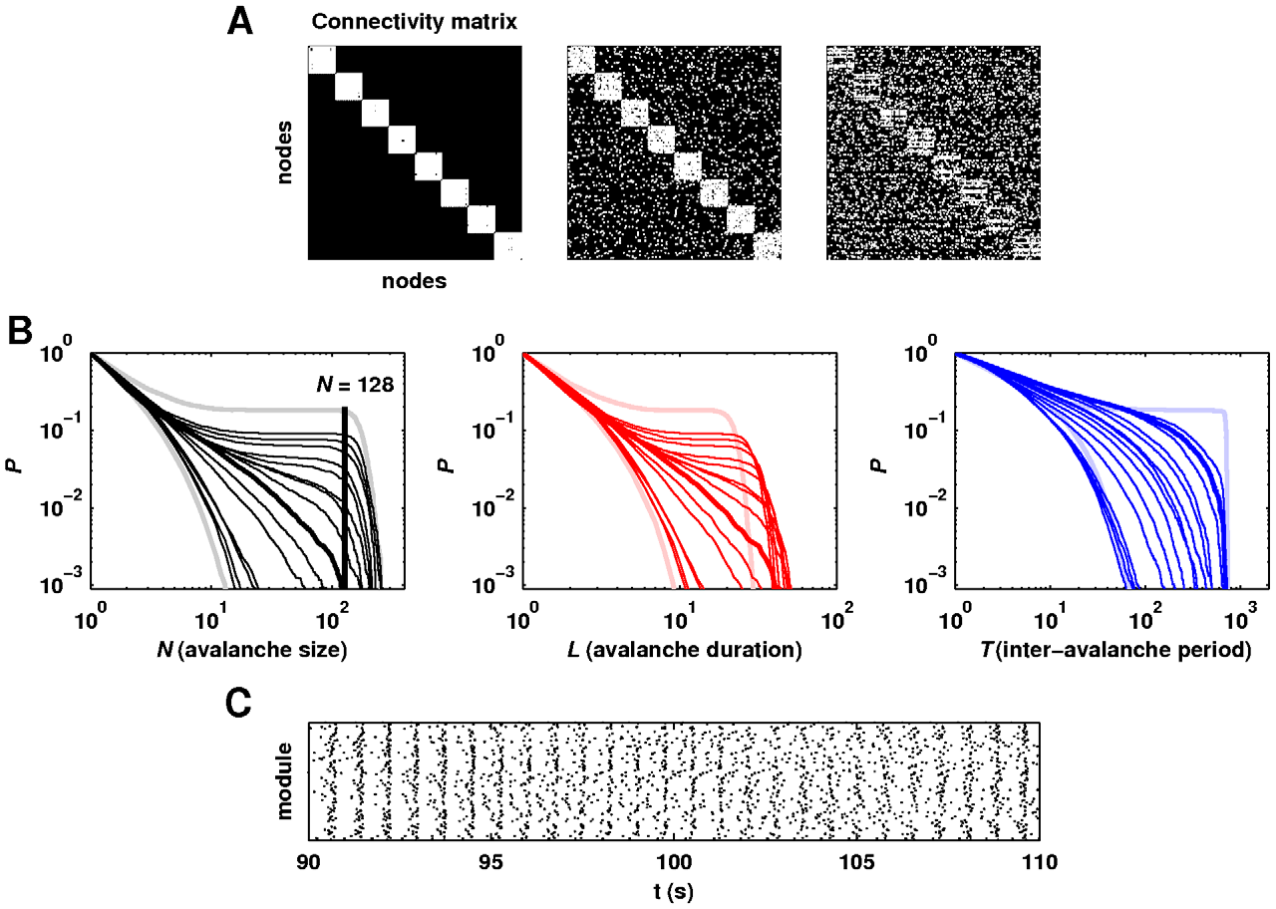
Phase transition from subcritical to supercritical network dynamics. (a) Illustrative ordered (left), intermediate (center) and random (right) nonhierarchical connectivity matrices. Nonhierarchical networks are characterized by a homogeneous density of between-module excitatory connections (Figure 2a, blue lines) (b) Cumulative probability distributions of avalanche sizes, avalanche durations and inter-avalanche intervals emergent on nonhierarchical networks. Subcritical dynamics (concave distributions) correspond to less randomized networks, supercritical dynamics (convex distributions) correspond to more randomized networks, while critical dynamics (linear-like distributions, in bold) occur between these two extremes. Gray, pink and light blue distributions correspond to random networks (concave distributions) and ordered networks (convex distributions). (c) An illustrative module spike raster of critical dynamics.

We initially examined dynamics emergent on nonhierarchical modular networks (Figure 4a). We gradually randomized these networks by rewiring excitatory connections in a way that increased the number of connections between modules. At one extreme, ordered nonhierarchical networks had no intermodule synapses. At the other extreme, random nonhierarchical networks had homogeneously distributed intra- and intermodule excitatory synapses. Between these two extremes, nonhierarchical networks had a varying number of homogeneously distributed intermodular excitatory synapses. The location of synapses in each network was fixed during simulations, but synaptic weights continuously fluctuated according to the STDP rule.

All nonhierarchical networks had a connectivity-independent neuron spike rate of 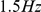, and a stable weight distribution (Figure 1b). In addition, these networks had module spike rates of 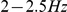. Ordered networks had no intermodular connections, and correspondingly showed subcritical uncoordinated dynamics. Random networks had large numbers of intermodular connections and correspondingly showed supercritical globally synchronous dynamics. A narrow range of network topologies between these two extremes was associated with critical dynamics, characterized by power-law distributions of avalanche sizes and durations (Figure 4b,c). Distributions of inter-avalanche intervals likewise changed from subcritical to supercritical, but did not follow consistent power laws at this transition (Figure 4b).

**Figure 5:**
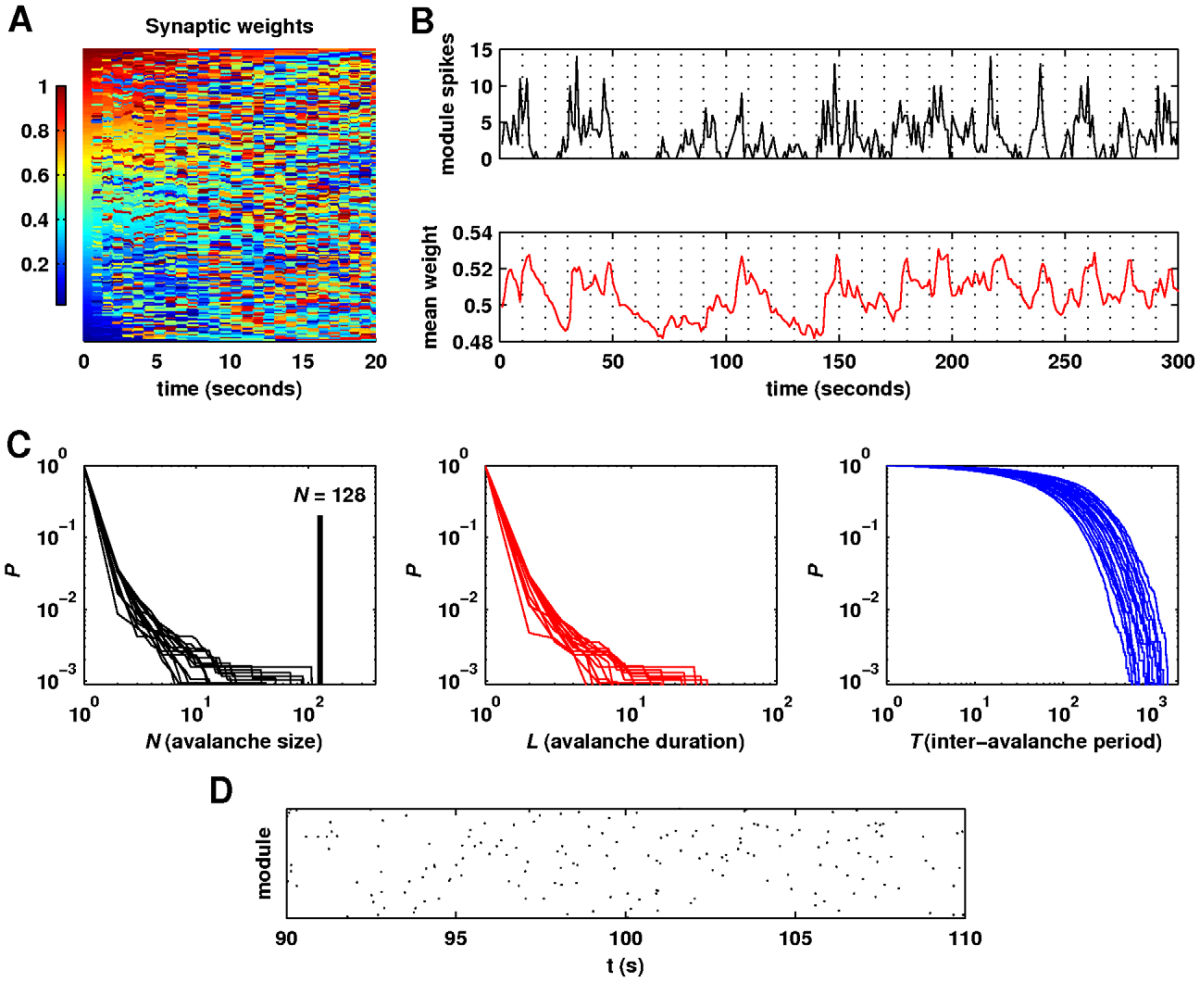
Relationship between spike-timing-dependent synaptic plasticity and network dynamics. (a) Fluctuations of within-module synaptic weights over a 20 second period. Synaptic weights were rank-ordered and assigned a rank-specific color at the first sampled time step. At subsequent steps, weights were re-ranked and therefore reordered, but the color-coding remained fixed. The mixing of colors hence represents fluctuations in rank positions. Stable synaptic weight distributions allowed the inference of weight fluctuations from these rank fluctuations. Weights were sampled at 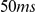 intervals. (b) Illustrative fluctuations in the number of module spikes (top) and in the mean within-module excitatory synaptic weights (bottom), recorded over a 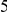 minute period from a single module. Module spikes were binned at 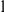 second intervals, and synaptic weights were sampled at 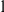 second intervals. (c) Cumulative probability distributions of avalanche sizes, avalanche durations and inter-avalanche intervals and (d) an illustrative module spike raster of dynamics emergent on nonhierarchical network topologies in Figure 4a, with frozen synaptic weights.

Despite the stable weight distributions, activity-dependent fluctuations in synaptic weights continuously occurred (Figure 5a,b). In order to investigate the impact of these fluctuations on global network dynamics, we examined the effect of freezing plasticity after five minutes of initial transient simulation. This procedure fixed the values of individual weights, and hence preserved the same neuronal spike rate of 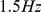. However, this procedure dramatically disrupted within-module neuronal synchrony: module spike rate dropped to less than 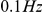 and dynamics on all networks became highly subcritical (Figure 5c,d). Module spike rate remained negligible despite increases in external current, and consequent increases in neuronal spike rate. Furthermore, module spike rate remained negligible with an even more stringent control condition, which allowed synaptic weight changes at spike times, but made these changes by randomly drawing weights from the distribution in Figure 1c, rather than according to the STDP rule (results not shown). On the other hand, as we show below, a change from soft to hard bounds in the STDP rule preserved equivalent dynamics, despite changing the weight distribution (Figure 1c–e). In addition, halving the STDP learning rate preserved equivalent dynamics when network size was doubled. Together, these findings indicate that the precise patterns of STDP-driven fluctuations enabled the formation of coherent within-module dynamics in our model.

### Hierarchical modularity and low wiring cost enabled a broad critical regime

**Figure 6:**
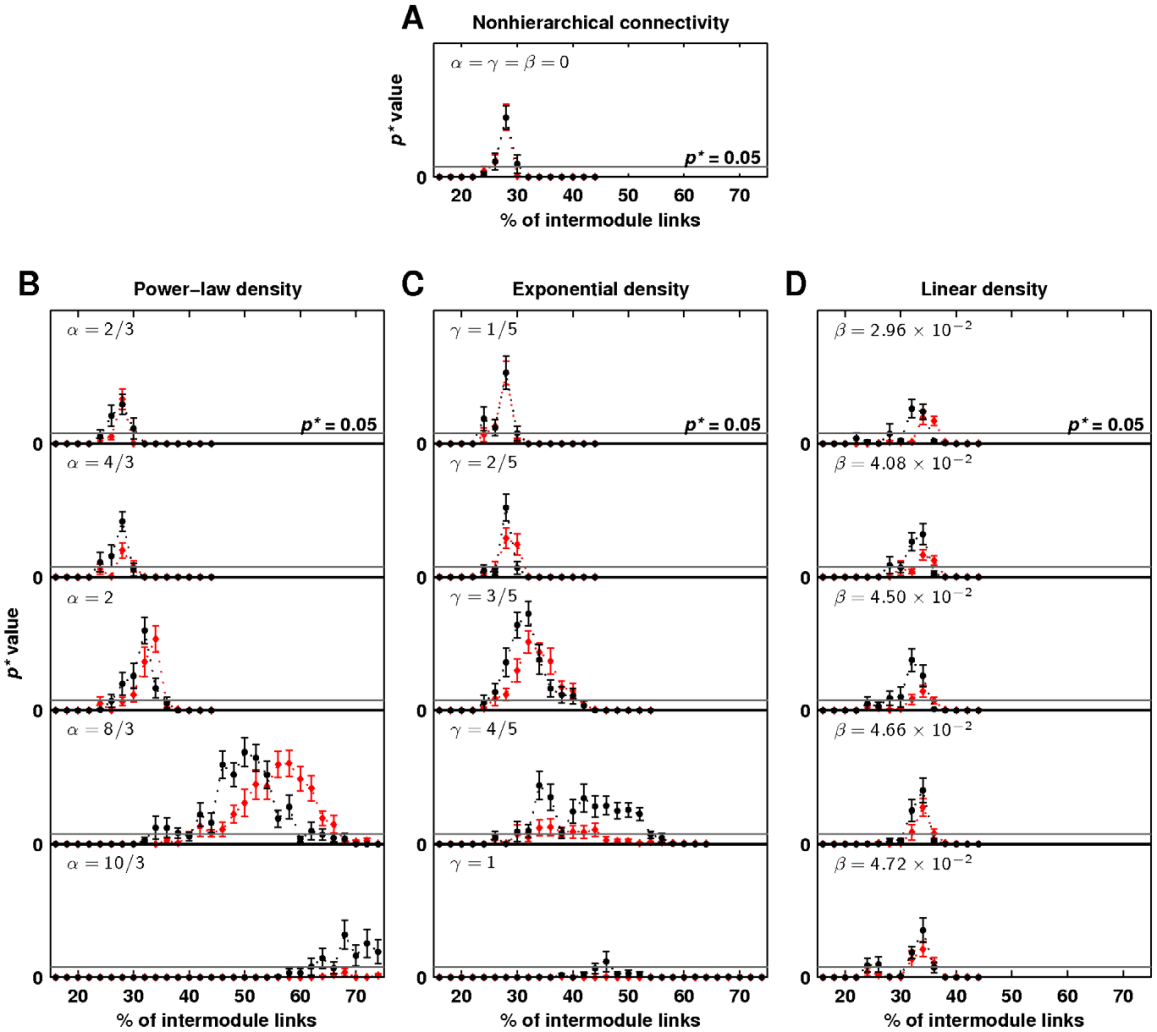
Relationship between hierarchical modularity, wiring cost and network dynamics. Statistical significance of power-law distributions of avalanche sizes (black) and durations (red) as a function of network randomization, for (a) nonhierarchical and hierarchical (b) power-law, (c) exponential and (d) linear density scaling functions. Gray lines show the 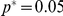 threshold for power-law scaling. Error bars represent the standard error of the mean from 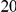 simulations.

Nonhierarchical connectivity is neurobiologically implausible, because of the high wiring cost associated with a large number of long-range connections, and because hierarchical modularity is evident in multiscale neuroanatomical organization [25]. We hence examined a more plausible connectivity by defining a framework in which connections were probabilistically placed within explicit spatial hierarchical levels, according to predefined power-law, exponential and linear scaling functions (see Methods and Figure 2). Figure 6 compares the critical regimes associated with nonhierarchical connectivity (Figure 6a), and with hierarchical power-law, exponential and linear (Figure 6b–d) connectivities. The rows in Figure 6b–d represent different wiring costs for each hierarchical organization. Most strikingly, low-cost power-law and exponential connectivities were associated with a broad critical regime. This regime was especially evident for the power-law connectivity with 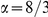 (fourth row in Figure 6b), as this was the only studied connectivity simultaneously associated with a broad regime of power-law distributed avalanche sizes and power-law distributed avalanche durations. Connectivities with higher wiring cost, such as all linear connectivities, showed narrow critical regimes. Connectivities with very low wiring cost did not show broad critical regimes, presumably because the numbers of long range connections in these connectivities were insufficient to enable the emergence of large events.

**Figure 7:**
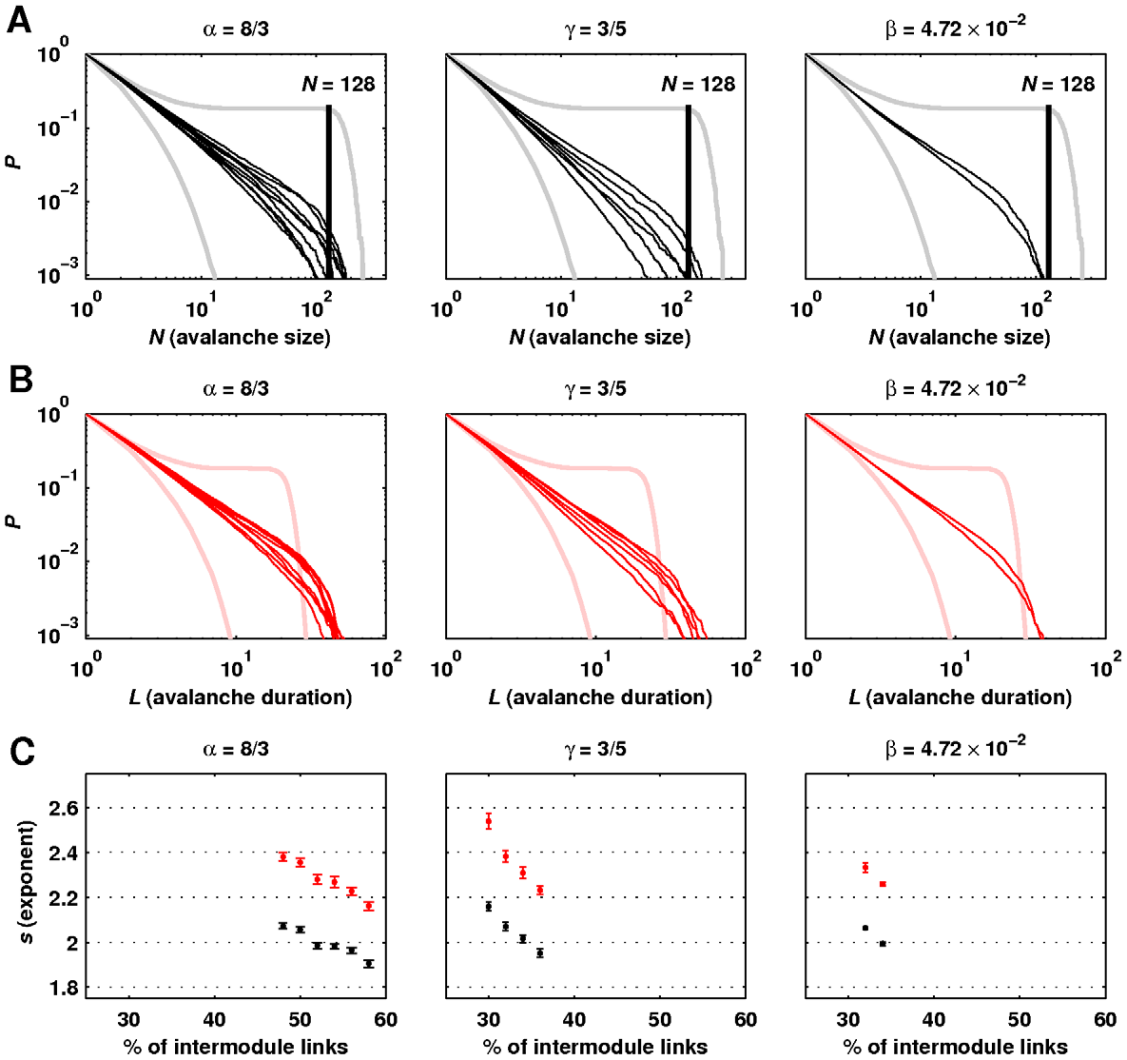
Illustrative power-law distributions of avalanche sizes and durations. Cumulative probability distributions of avalanche (a) sizes and (b) durations for the optimal power-law (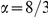), exponential (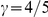) and linear (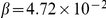) density scaling functions. Gray and pink distributions correspond to random networks (concave distributions) and ordered networks (convex distributions). (c) Mean exponents of statistically significant power-law distributions of avalanche sizes (black) and durations (red), as a function of network randomization. Error bars represent the standard error of the mean from 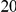 simulations.

Figure 7a,b shows statistically significant power-law distributions of avalanche sizes and durations for the optimal power-law, exponential and linear connectivities. The greater number of power-law distributions for the power-law and exponential connectivities, compared with linear connectivity, is clearly visible. Figure 7c illustrates the values of power-law exponents for connectivities in which avalanche sizes and durations simultaneously followed statistically significant power laws. Exponents of avalanche size distributions associated with power-law connectivities were close to 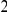 and hence accurately resembled empirically estimated exponents of neuronal avalanche size distributions at the same bin size [5], [14]. Exponents decreased with increasing network randomization.

**Figure 8:**
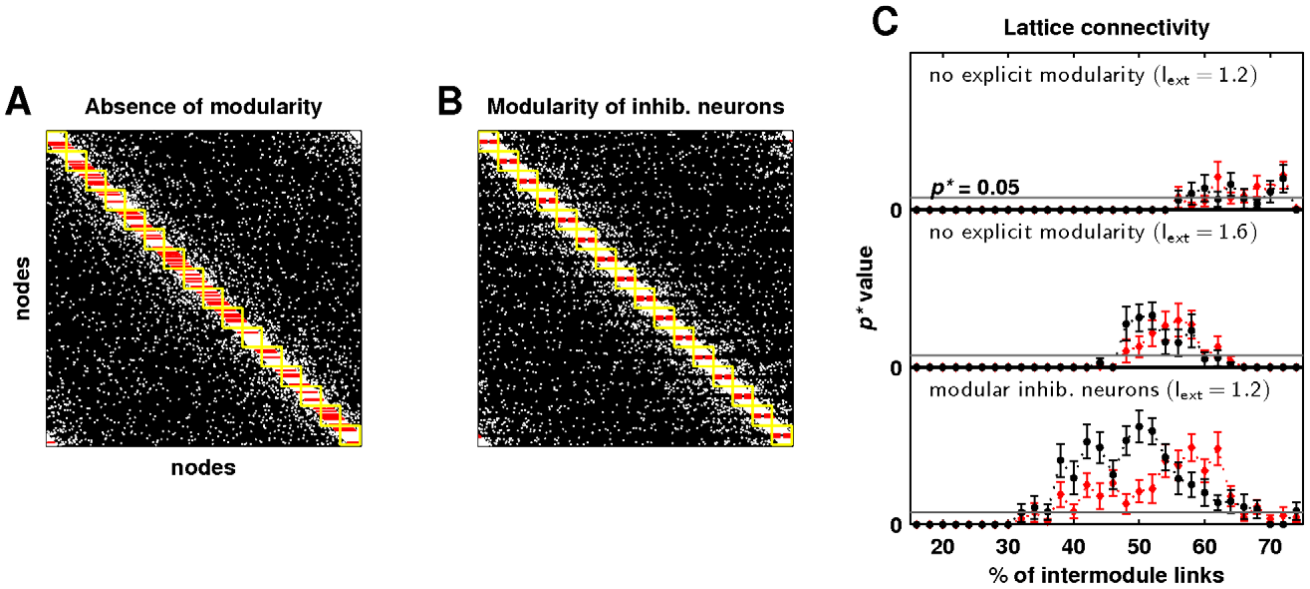
Role of modularity and low wiring cost in emergence of self-organized critical network dynamics. (a) An illustrative lattice connectivity matrix with the optimal power-law (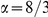) density scaling function. Presumed network modules are shown in yellow, while inhibitory synapses are shown in red. Note the presence of significant numbers of intermodule inhibitory synapses. (b) A variant of the connectivity matrix in (a), with modularity of inhibitory neurons. (c) Statistical significance of power-law distributions of avalanche sizes (black) and durations (red) for the lattice (top), the lattice with restored module spike rate (center), and the modified lattice, in which inhibitory neurons were arranged into explicit modules. Gray lines show the 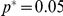 threshold for power-law scaling. Error bars represent the standard error of the mean from 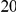 simulations.

We sought to disambiguate the association between modularity and the broad critical regime by examining dynamics emergent on lattices with optimal power-law connectivity, but no explicit modular structure (Figure 8a). For this purpose, we constructed lattices of the same size and degree as the hierarchical connectivity networks, and we randomized these lattices by distributing off-diagonal connections according to the power-law density scaling function with 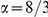. In this way, we could focus on the effect of hierarchical modularity by retaining most other features of original network organization, including wiring cost. Dynamics on these lattice networks had substantially reduced module spike rates (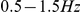) and were associated with a rapid phase transition and a loss of the broad critical regime (Figure 8c, top). An increase in external current restored the original module spike rate of 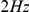 and consequently broadened the critical regime, although not to the original level (Figure 8c, middle). On the other hand, when modularity was implicitly reintroduced by rearranging inhibitory synapses into modules (Figure 8b), a broad critical regime reappeared without changes in external current (Figure 8c, bottom). These findings suggest that modularity of inhibitory connections facilitated coherent within-module dynamics.

**Figure 9:**
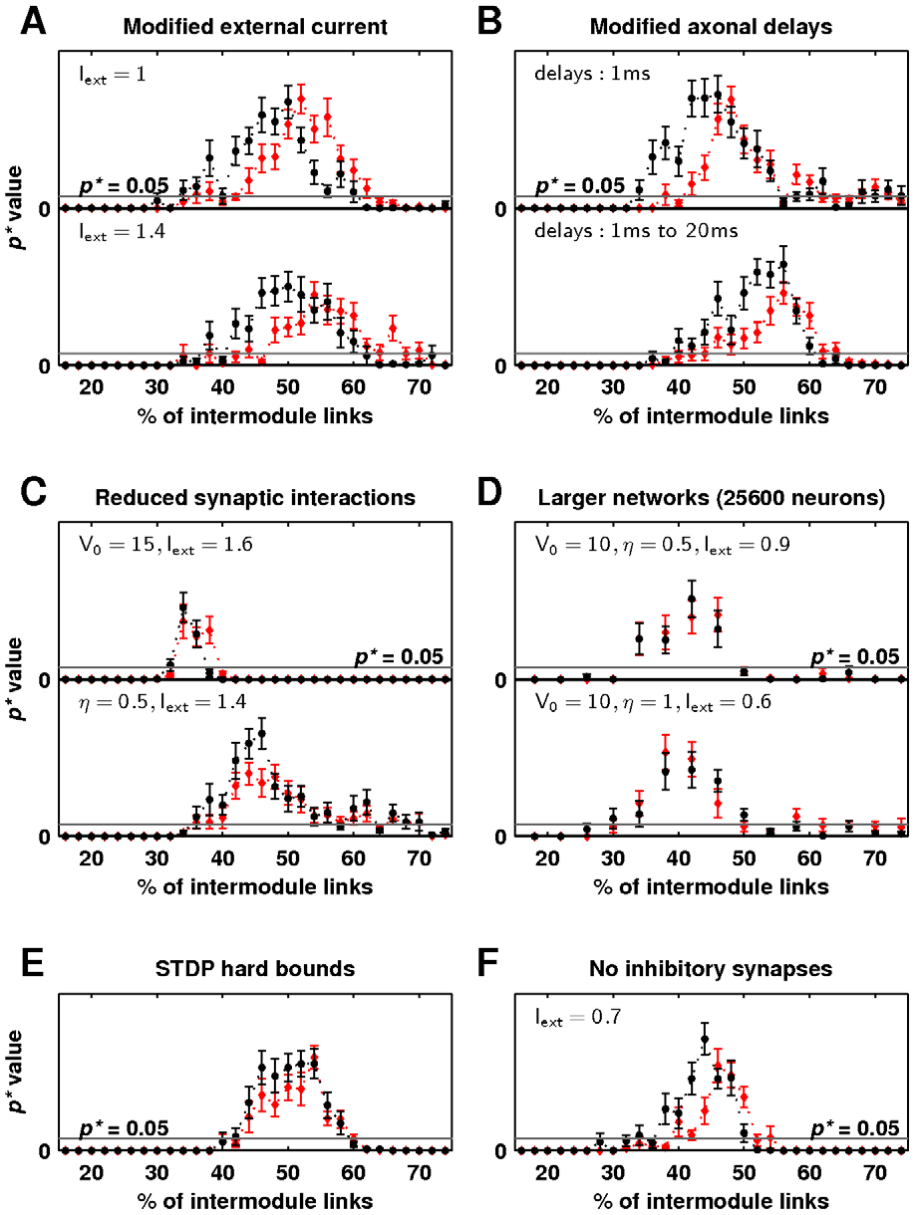
Robustness of self-organized critical network dynamics. Statistical significance of power-law distributions of avalanche sizes (black) and durations (red) as a function of network randomization for the optimal power-law (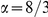) density scaling function associated with (a) changes in external current (default 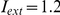), (b) changes in conduction delays (default delays are uniformly distributed between 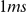 and 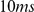), (c) weaker postsynaptic response (default 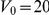) and slower STDP learning rate (default 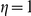) (d) doubling of module size to 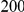 neurons and network size to 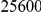 neurons and reductions in postsynaptic response, STDP learning rate and external current (e) changes from soft to hard STDP weight bounds, and (f) removal of inhibitory synapses. Gray lines show the 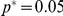 threshold for power-law scaling. Error bars represent the standard error of the mean from 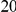 simulations.

We explored robustness of the broad critical regime (for the optimal power-law density scaling function) to other meaningful changes in neurobiologically relevant parameters, such as changes in external current, changes in conduction delays, changes in the postsynaptic response, presence of neuronal inhibition, changes in the STDP rule and changes in network size (Figure 9). Theoretically, self-organized criticality emerges in systems with low external drive and strong internal interactions, and the responses of our model to variation of parameters were meaningful in this context. It is worth noting that we assessed the strength of external drive by the associated neuronal spike rate. Specifically, we considered the external current of 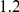 to represent a low external drive even though this value substantially exceeds the minimal value of 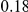 required to sustain neuronal activity (see Text S1 for details). In our simulations the broad critical regime was robust to moderate variations of external current and delays (Figure 9a,b), but began to disappear when external current exceeded 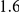 (as external drive became too strong), or when delay lengths were quadrupled to the range of 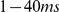 (as internal interactions lost spike precision). The regime was narrowed when the postsynaptic response weakened (Figure 9c, top), but was preserved when the STDP learning rate was reduced (Figure 9c, bottom). In both cases, we controlled for changes in neuronal spike rate by increasing external current. The regime was broadened by a stronger postsynaptic response and by a higher STDP learning rate (results not shown, as the associated parameter values are unrealistically high).

We hypothesized that our network models required strong postsynaptic responses and fast STDP learning rates to compensate for the small number of synaptic connections of each neuron. Excitatory neurons in our model connected with only 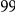 other neurons, while *in vivo* each neuron is thought to have thousands of synapses. We compensated for the small number of connections in our model by setting the postsynaptic-response magnitude of each neuron to a value which could theoretically exceed the neuron spike threshold and by using an instantaneous STDP learning rate that substantially exceeds empirically observed values (Table 1). When we doubled our module size to 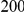 neurons, and consequently doubled our network size to 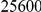 neurons, we were able to simultaneously halve the values of postsynaptic-response magnitudes and STDP learning rates and hence bring these values much closer to empirically observed values [49]. Specifically, the broad critical regime in these larger networks was preserved when the postsynaptic-response magnitude was halved, the STDP learning rate was halved, and the external current was reduced from 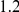 to 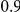 (Figure 9d, top). Alternatively, the regime was preserved when the postsynaptic-response magnitude was halved, the STDP learning rate remained unchanged, and the external current was halved (Figure 9d, bottom). These findings show that realistically large numbers of synaptic connections are likely to facilitate strong internal interactions in the presence of biologically realistic parameter values.

In addition to these variations, the broad critical regime did not qualitatively change when inhibitory synapses were removed, provided the loss of inhibition was controlled by reductions in external current (Figure 9e). The broad critical regime was likewise preserved when soft weight bounds were changed to hard weight bounds in the STDP rule (Figure 9f).

## Discussion

Despite increasing theoretical support and empirical evidence for critical brain dynamics, most models of these dynamics have been fairly abstract, and have largely not considered the influence of neuroanatomically realistic determinants. In this study, we employed a realistic model of neuronal network dynamics, and discerned an association between modularity, low cost of wiring, spike-timing-dependent synaptic plasticity, and a dynamical regime indicative of self-organized criticality. We hence discerned an intriguing and novel association between multiple neurobiological features of complex brain structure and dynamics, including self-similarity of structure (power-law connectivity) and self-similarity of dynamics (self-organized criticality). We now discuss the mechanisms behind this association, and the implication of our findings for empirical research.

### Synaptic plasticity and neuronal ensemble synchronization

We found that despite seemingly stable neuronal activity, spike-timing-dependent plasticity enabled coherent within- and between-module neuronal activity. Furthermore, we showed that two variations of the STDP rule produced distinct weight distributions, but enabled a broad critical regime on conducive network topologies. In contrast, fixed or randomly altered synaptic weights were associated with subcritical dynamics and negligible module spike rates. STDP may facilitate coherent within-module activity by intermittently potentiating and depressing synapses between reciprocally connected neurons. In small networks, simulations showed that intermittent synaptic potentiation and depression was associated with pairwise neuronal synchrony, fluctuations of synaptic weights and continuous reversal of phase differences between reciprocally connected pairs of neurons (results not shown). In our networks, within-module weights were potentiated during module spikes, and depressed between module spikes (Figure 5b). These activity-dependent fluctuations hence clearly played an important role in facilitating neuronal ensemble synchronization.

Recent studies have shown the importance of short-term synaptic depression in self-organized critical dynamics in networks of spiking neurons, but have not concurrently considered the effects of STDP [33], [35]. Our study illustrates the importance of STDP in self-organization and hence provides a alternative generative model of critical dynamics in networks of spiking neurons. A principled comparison of the role of these two forms of plasticity in self-organized criticality is hence an important subject of future research. The distinct mechanism of these forms of plasticity may also allow to disambiguate their role empirically with pharmacological manipulations in real neuronal systems.

### Modularity, low wiring cost and self-organized criticality

Modular networks with low wiring cost showed a broad critical regime. Modular networks with high wiring cost showed a narrow critical regime, possibly due to high numbers of costly long-range connections, which enabled a rapid onset of globally synchronous, supercritical dynamics. Lattice networks with low wiring cost showed a narrowed critical regime due to uncoordinated inhibition and a consequent loss of coherent ensemble dynamics. Modularity and low wiring cost were hence simultaneously required for self-organized criticality to emerge. This simultaneous requirement is notable, as both properties are thought to be ubiquitously present in neuroanatomical organization.

### Dependence on parameters and other neurobiological features

In an early comprehensive exposition, Jensen [4] addressed the potentially confusing meaning of self-organization to criticality: “[s]elf-organization to criticality will definitely occur only under certain conditions; one will always be able to generalize a model sufficiently to lose the critical behavior. Hence the question becomes just what is relevant in a given context. This is where a super-general approach must be supplemented by insight from the specific science to which a given system belongs.” In this spirit, we examined neurobiologically meaningful variations in parameters such as external current and conduction delays. We found that the broad critical regime was generally preserved despite variations of these parameters and, consequently, finetuning was not required for self-organized critical dynamics to emerge. More specifically, strong synaptic interactions with low external current (i.e. short delays, strong postsynaptic responses, high STDP learning rate) favored a broad critical regime, while weak synaptic interactions with high external current (i.e. long delays, weak postsynaptic response, low STDP learning rate) favored a narrow critical regime. These findings indicate that critical dynamics primarily emerged through internal interactions, rather than external drive. The findings hence provide further evidence for the self-organizing nature of the observed dynamics. The strong postsynaptic response and STDP learning rate in our model compensated for the relatively low synaptic connectivity, and could be markedly lowered in larger networks without detriment to the broad critical regime.

We found that inhibitory neurons in our model did not explicitly enable a broad critical regime. In contrast, recent network simulations of simple stochastic neurons by Benayoun et al. [50] show that inhibitory neurons enable self-organized criticality by balancing the network. However, the differences in neuronal dynamics, and the absence of statistically significant power laws in the Benayoun et al. study, make it difficult to directly compare our findings. We do show however, that the presence of inhibitory neurons in our networks was compatible with self-organized critical dynamics only if these neurons were organized in modules. These modules correspond to realistic local inhibitory connectivity, rather than the less realistic long-range inhibitory connectivity. Inhibitory neurons may also play a more prominent role in other types of network dynamics, such as oscillations.

### Implications for empirical research

Our findings may be used to generate empirically testable hypotheses of the relationship between anatomical connectivity and emergent network dynamics. For instance, we hypothesize that self-organized critical dynamics in dissociated neuronal cultures emerge on a low-cost modular neuroanatomical connectivity. Recent studies show that dissociated neuronal cultures self-organize towards a critical state, via subcritical and supercritical states [12]–[13], [51]. Cultured dissociated neurons self-organize by forming axonal and dendritic arborizations, and synaptic connections [44]. In the first week of culture, self-organization is non-activity-dependent, and may show preference towards spatial proximity. After the first week of culture, the network becomes spontaneously active, and self-organization becomes activity-dependent.

Our findings may hence be used to explicitly compare structure and dynamics of dissociated neuronal cultures, throughout this period of self-organization. A recent study found that functional activity patterns of dissociated neuronal cultures constitute a small-world network [52]. Novel methods of network reconstruction from avalanche dynamics [53] may allow to study structural network properties of these cultures. For instance, future empirical work may study the relationship between specific anatomical measures (e.g. wiring cost) and dynamical measures (e.g. exponent values of power-law distributions) in such networks, throughout self-organization. Alternatively, it may be possible to study dynamics in real neuronal networks with externally controlled anatomical connectivity [54].

### Limitations and methodological considerations

A clear limitation of our study is the oversimplified symmetric hierarchical organization and the relatively small size of our model. Substantial increases in the number of modules, and in the number of neurons within modules, are required to make realistic inferences about neuronal dynamics at larger scales. The study hence sets the groundwork for simulations of large networks of spiking neurons and for characterization of spatiotemporal activity patterns emergent on these networks. Such simulations may be conducted on increasingly detailed maps of large-scale anatomical connectivity in healthy subjects [55]–[57] and in subjects with connectivity disorders, such as Alzheimer's disease [58] and schizophrenia [59]. These simulations will be the subject of future studies.

Studies of neuronal dynamics often employ numerical integration schemes (such as the Euler method), and manually store all previous spike times to compute synaptic currents. An advantage of the integrate-and-fire neuron model is the ability to integrate subthreshold activity exactly and incorporate effects of all previous spikes without the need for explicit summation at each step [60]. In addition, interpolation of spike times between time steps avoids artefactual synchrony and is especially important in simulations with spike-timing-dependent plasticity. Hence, while our results remain subject to numerical error, the particular integration scheme we employ [42] substantially reduces the possibility of numerical artefacts.

Despite growing empirical evidence for self-organized criticality, several important studies argue against this evidence, by either noting the potential for spurious reports of power-law scaling, or by attributing such scaling to simpler mechanisms, such as diffusive processes [47], [61]–[62]. Two observations favor the presence of self-organized criticality in our model. Firstly, we estimate power-law scaling with rigorous statistical tests [36], rather than the more commonly used unreliable linear least-squares-based methods. We use a method with very high specificity and we can hence be highly certain that the detected power-law distributions are genuine. On the other hand, the method may have potentially low sensitivity, and may hence underestimate the presence of power laws in our data. Secondly, we find that these power-law distributions are associated with a phase transition, suggesting that dynamics evolve at the critical point. In addition, we note that it is not straightforward to compare findings between studies that focus on different scales and types of neuronal activity. Hence, while much evidence for critical brain dynamics comes from studies of low frequency spatiotemporal dynamics (as in this study), these dynamics cannot be trivially related to other phenomena, such as noise-like processes in recordings of high frequency neurophysiological signals [62].

In conclusion, we show an association between modularity, low cost of wiring, synaptic plasticity and self-organized criticality in a neurobiologically realistic model of neuronal activity. Our findings theoretically reinforce the reciprocal relationship between connectivity and dynamics on multiple spatial scales.

## Supporting Information

Text S1 Supporting information code.
(PDF)

Text S2 Supporting information code.
(TXT)

Text S3 Supporting information code.
(TXT)

Text S4 Supporting information code.
(TXT)

Text S5 Supporting information code.
(TXT)

Text S6 Supporting information code.
(TXT)

Text S7 Supporting information code.
(TXT)

Text S8 Supporting information code.
(TXT)

Text S9 Supporting information code.
(TXT)

Text S10 Supporting information code.
(TXT)

Text S11 Supporting information code.
(TXT)

Text S12 Supporting information code.
(TXT)

Text S13 Supporting information code.
(TXT)

Text S14 Supporting information code.
(TXT)

Text S15 Supporting information code.
(TXT)

Text S16 Supporting information code.
(TXT)

Text S17 Supporting information code.
(TXT)

## References

[1] Plenz D, Thiagarajan TC (2007). The organizing principles of neuronal avalanches: cell assemblies in the cortex?. Trends Neurosci.

[2] Beggs JM (2008). The criticality hypothesis: how local cortical networks might optimize information processing.. Philos Trans R Soc Lond A.

[3] Bak P, Tang C, Wiesenfeld K (1988). Self-organized criticality.. Phys Rev A.

[4] Jensen H (1998). Self-Organized Criticality: Emergent Complex Behavior in Physical and Biological Systems (Cambridge Lecture Notes in Physics).

[5] Beggs JM, Plenz D (2003). Neuronal avalanches in neocortical circuits.. J Neurosci.

[6] Beggs JM, Plenz D (2004). Neuronal avalanches are diverse and precise activity patterns that are stable for many hours in cortical slice cultures.. J Neurosci.

[7] Haldeman C, Beggs JM (2005). Critical branching captures activity in living neural networks and maximizes the number of metastable States.. Phys Rev Lett.

[8] Shew WL, Yang H, Yu S, Roy R, Plenz D (2011). Information Capacity and Transmission Are Maximized in Balanced Cortical Networks with Neuronal Avalanches.. J Neurosci.

[9] Kinouchi O, Copelli M (2006). Optimal dynamical range of excitable networks at criticality.. Nat Phys.

[10] Shew WL, Yang H, Petermann T, Roy R, Plenz D (2009). Neuronal Avalanches Imply Maximum Dynamic Range in Cortical Networks at Criticality.. J Neurosci.

[11] de Arcangelis L, Herrmann HJ (2010). Learning as a phenomenon occurring in a critical state.. Proc Natl Acad Sci U S A.

[12] Pasquale V, Massobrio P, Bologna LL, Chiappalone M, Martinoia S (2008). Self-organization and neuronal avalanches in networks of dissociated cortical neurons.. Neuroscience.

[13] Tetzlaff C, Okujeni S, Egert U, Wörgötter F, Butz M (2010). Self-Organized Criticality in Developing Neuronal Networks.. PLoS Comput Biol.

[14] Petermann T, Thiagarajan TC, Lebedev MA, Nicolelis MA, Chialvo DR (2009). Spontaneous cortical activity in awake monkeys composed of neuronal avalanches.. Proc Natl Acad Sci U S A.

[15] Linkenkaer-Hansen K, Nikouline VV, Palva JM, Ilmoniemi RJ (2001). Long-range temporal correlations and scaling behavior in human brain oscillations.. J Neurosci.

[16] Stam CJ, de Bruin EA (2004). Scale-free dynamics of global functional connectivity in the human brain.. Hum Brain Mapp.

[17] Kitzbichler MG, Smith ML, Christensen SR, Bullmore E (2009). Broadband criticality of human brain network synchronization.. PLoS Comput Biol.

[18] He BJ, Zempel JM, Snyder AZ, Raichle ME (2010). The temporal structures and functional significance of scale-free brain activity.. Neuron.

[19] Honey CJ, Kötter R, Breakspear M, Sporns O (2007). Network structure of cerebral cortex shapes functional connectivity on multiple time scales.. Proc Natl Acad Sci U S A.

[20] Honey CJ, Sporns O, Cammoun L, Gigandet X, Thiran JP (2009). Predicting human resting-state functional connectivity from structural connectivity.. Proc Natl Acad Sci U S A.

[21] Ghosh A, Rho Y, McIntosh A, Kötter R, Jirsa V (2008). Cortical network dynamics with time delays reveals functional connectivity in the resting brain.. Cogn Neurodyn.

[22] Deco G, Jirsa V, McIntosh AR, Sporns O, Kötter R (2009). Key role of coupling, delay, and noise in resting brain fluctuations.. Proc Natl Acad Sci U S A.

[23] Humphries MD, Gurney K, Prescott TJ (2006). The brainstem reticular formation is a small-world, not scale-free, network.. Proc Biol Sci.

[24] Chen BL, Hall DH, Chklovskii DB (2006). Wiring optimization can relate neuronal structure and function.. Proc Natl Acad Sci U S A.

[25] Bassett DS, Greenfield DL, Meyer-Lindenberg A, Weinberger DR, Moore SW (2010). Efficient physical embedding of topologically complex information processing networks in brains and computer circuits.. PLoS Comput Biol.

[26] Bullmore E, Barnes A, Bassett DS, Fornito A, Kitzbichler M (2009). Generic aspects of complexity in brain imaging data and other biological systems.. Neuroimage.

[27] Markram H, Tsodyks M (1996). Redistribution of synaptic efficacy between neocortical pyramidal neurons.. Nature.

[28] Song S, Miller KD, Abbott LF (2000). Competitive Hebbian learning through spike-timing-dependent synaptic plasticity.. Nat Neurosci.

[29] Caporale N, Dan Y (2008). Spike-timing-dependent plasticity: a Hebbian learning rule.. Annu Rev Neurosci.

[30] Rubinov M, Sporns O, van Leeuwen C, Breakspear M (2009). Symbiotic relationship between brain structure and dynamics.. BMC Neurosci.

[31] de Arcangelis L, Perrone-Capano C, Herrmann HJ (2006). Self-Organized Criticality Model for Brain Plasticity.. Phys Rev Lett.

[32] Pellegrini GL, de Arcangelis L, Herrmann HJ, Perrone-Capano C (2007). Activity-dependent neural network model on scale-free networks.. Phys Rev E.

[33] Levina A, Herrmann JM, Geisel T (2007). Dynamical synapses causing self-organized criticality in neural networks.. Nat Phys.

[34] Vertes PE, Duke T (2010). Effect of network topology on neuronal encoding based on spatiotemporal patterns of spikes.. HFSP J.

[35] Millman D, Mihalas S, Kirkwood A, Niebur E (2010). Self-organized criticality occurs in non-conservative neuronal networks during ‘up’ states.. Nat Phys.

[36] Clauset A, Shalizi CR, Newman MEJ (2009). Power-Law Distributions in Empirical Data.. SIAM Rev.

[37] Thivierge JP, Cisek P (2008). Nonperiodic synchronization in heterogeneous networks of spiking neurons.. J Neurosci.

[38] Swadlow HA (1985). Physiological properties of individual cerebral axons studied in vivo for as long as one year.. J Neurophysiol.

[39] Song S, Sjöström PJ, Reigl M, Nelson S, Chklovskii DB (2005). Highly nonrandom features of synaptic connectivity in local cortical circuits.. PLoS Biol.

[40] Maslov S, Sneppen K (2002). Specificity and stability in topology of protein networks.. Science.

[41] Watts DJ, Strogatz SH (1998). Collective dynamics of ‘small-world’ networks.. Nature.

[42] Morrison A, Straube S, Plesser HE, Diesmann M (2007). Exact subthreshold integration with continuous spike times in discrete-time neural network simulations.. Neural Comput.

[43] Eytan D, Marom S (2006). Dynamics and effective topology underlying synchronization in networks of cortical neurons.. J Neurosci.

[44] van Pelt J, Vajda I, Wolters PS, Corner MA, Ramakers GJA (2005). Dynamics and plasticity in developing neuronal networks in vitro.. Prog Brain Res.

[45] Sutherland GR, McNaughton B (2000). Memory trace reactivation in hippocampal and neocortical neuronal ensembles.. Curr Opin Neurobiol.

[46] Bauke H (2007). Parameter estimation for power-law distributions by maximum likelihood methods.. Eur Phys J B.

[47] Touboul J, Destexhe A (2010). Can Power-Law Scaling and Neuronal Avalanches Arise from Stochastic Dynamics?. PLoS ONE.

[48] Vuong Q (1989). Likelihood Ratio Tests for Model Selection and Non-Nested Hypotheses.. Econometrica.

[49] Morrison A, Aertsen A, Diesmann M (2007). Spike-Timing-Dependent Plasticity in Balanced Random Networks.. Neural Comput.

[50] Benayoun M, Cowan JD, van Drongelen W, Wallace E (2010). Avalanches in a Stochastic Model of Spiking Neurons.. PLoS Comput Biol.

[51] Stewart CV, Plenz D (2008). Homeostasis of neuronal avalanches during postnatal cortex development in vitro.. J Neurosci Methods.

[52] Srinivas KV, Jain R, Saurav S, Sikdar SK (2007). Small-world network topology of hippocampal neuronal network is lost, in an in vitro glutamate injury model of epilepsy.. Eur J Neurosci.

[53] Pajevic S, Plenz D (2009). Efficient Network Reconstruction from Dynamical Cascades Identifies Small-World Topology of Neuronal Avalanches.. PLoS Comput Biol.

[54] Feinerman O, Rotem A, Moses E (2008). Reliable neuronal logic devices from patterned hippocampal cultures.. Nat Phys.

[55] Sporns O, Tononi G, Kötter R (2005). The human connectome: A structural description of the human brain.. PLoS Comput Biol.

[56] Hagmann P, Cammoun L, Gigandet X, Meuli R, Honey CJ (2008). Mapping the structural core of human cerebral cortex.. PLoS Biol.

[57] Gong G, He Y, Concha L, Lebel C, Gross DW (2009). Mapping anatomical connectivity patterns of human cerebral cortex using in vivo diffusion tensor imaging tractography.. Cereb Cortex.

[58] He Y, Chen Z, Evans A (2008). Structural Insights into Aberrant Topological Patterns of Large-Scale Cortical Networks in Alzheimer's Disease.. J Neurosci.

[59] Bassett DS, Bullmore E, Verchinski BA, Mattay VS, Weinberger DR (2008). Hierarchical Organization of Human Cortical Networks in Health and Schizophrenia.. J Neurosci.

[60] Plesser HE, Diesmann M (2009). Simplicity and efficiency of integrate-and-fire neuron models.. Neural Comput.

[61] Bedard C, Kroger H, Destexhe A (2006). Does the 1/f frequency scaling of brain signals reflect self-organized critical states?. Phys Rev Lett.

[62] Miller KJ, Sorensen LB, Ojemann JG, den Nijs M (2009). Power-law scaling in the brain surface electric potential.. PLoS Comput Biol.

