# Supplementary material for: Neurobiologically Realistic Determinants of Self-Organized Criticality in Networks of Spiking Neurons
Source: PLoS Comput Biol. 2011 Jun 2;7(6):e1002038. doi: 10.1371/journal.pcbi.1002038 (PMC3107249; doi:10.1371/journal.pcbi.1002038)
Supplement: Text S1 — Supporting information code. (PDF) [file pcbi.1002038.s001.pdf]

Neurobiologically realistic determinants of self-organized criticality in networks of spiking neurons: Supplementary information.

Mikail Rubinov, Olaf Sporns, J.-P. Thivierge, Michael Breakspear

Summary

We summarize an accurate and efficient scheme for simulating large networks of integrate-and-fire neurons. We adapted this scheme from Morrison et al. (2007). The scheme consists of two parts:

1. Exact computation of subthreshold membrane potentials on discrete time points, in parallel.

2. Estimation of continuous spike times by interpolation between discrete time points. This estimation is especially important in our study, because STDP is highly dependent on precise spike timing.

Background: the integrate-and-fire model

In a network of integrate-and-fire neurons, the membrane potential for a neuron is given by

$$C\frac{dV}{dt} = I_{\text{leak}}(t) + I_{\text{syn}}(t) + I_{\text{ext}}, \tag{1}$$

where C is membrane capacitance and I_{leak}, I_{syn}, I_{ext} are currents (Burkitt et al., 2006).

I_{leak} represents passive current leaks across the membrane and is given by

$$I_{\text{leak}}(t) = -g(V - E),$$

where g is the membrane conductance, and E is the equilibrium potential.

I_{syn} represents currents generated by synaptic inputs, and is given by

$$I_{\text{syn}}(t) = V_0 \sum_i w_i \sum_{t_i} \left(e^{-(t-t_i)/\tau_1} - e^{-(t-t_i)/\tau_2}\right), \tag{2}$$

where V_0 is a constant, w_i is the weight of presynaptic neuron i, t_i are the times of previous spikes of i ($t_i < t$), and τ_1 and τ_2 are synaptic decay constants.

I_{ext} represents the external current and needs to be kept constant. Simulations with variable I_{ext} are hence composed of multiple simulation segments, with constant I_{ext} in each segment.

Exact subthreshold integration of the integrate-and-fire model

Exact integration without synaptic currents

Without synaptic currents, (1) is a one-dimensional system,

$$C\frac{dV}{dt} = I_{\text{leak}}(t) + I_{\text{ext}} = -g(V - E) + I_{\text{ext}} = -g\left(V - E - \frac{I_{\text{ext}}}{g}\right).$$

By substituting $y_1 = V - E - I_{\text{ext}}/g$, we obtain

$$C\frac{dV}{dt} = C\frac{dy_1}{dt} = -gy_1,$$

which is solved exactly as

$$y_1(t) = e^{-gt/C}y_1(0). \tag{3}$$

Henceforth we consider the evolution of y_1, noting that given our parameter values (Table 1), we may always revert to V via

$$V(t) = y_1(t) + E + \frac{I_{\text{ext}}}{g} = y_1(t) + 100I_{\text{ext}}.$$

Eq. (3) shows that y_1 exponentially decays to zero from $y_1(0) < 0$. Larger I_{ext} make $y_1(0)$ more negative, and hence increase the decay rate (somewhat like stretching a spring increases the speed of its subsequent compression). It follows that the membrane potential V may only cross its spike threshold V_{thr}, when the corresponding threshold $y_1^{\text{thr}} = V_{\text{thr}} - E - I_{\text{ext}}/g$ is negative. For our parameter values (Table 1) this requires $I_{\text{ext}} > 0.18$.

Given some initial condition $y_1(t)$ and a time step h, the constant $P(h) = e^{-gh/C}$ exactly computes $y_1(nh)$ for any integer n,

$$\begin{aligned} y_1(h) &= P(h)y_1(0) \\ y_1(2h) &= P(h)y_1(h) = P(h)^2 y_1(0) \\ y(nh) &= P(h)y_1((n-1)h) = P(h)^n y_1(0). \end{aligned} \tag{4}$$

Note that $P(h)$ needs to be calculated only once.

Exact integration with synaptic currents

Incorporation of synaptic currents makes (1) three dimensional. We now introduce two further auxiliary variables, y_2 and y_3, such that

$$y_2 = e^{-(t-t_j)/\tau_1} \qquad y_3 = e^{-(t-t_j)/\tau_2}$$

$$\tau_1 \frac{dy_2}{dt} = -y_2 \qquad \tau_2 \frac{dy_3}{dt} = -y_3. \tag{5}$$

At the arrival of each spike from neuron i, y_2 and y_3 are modified as

$$y_2 \leftarrow y_2 + V_0 w_i \qquad y_3 \leftarrow y_3 + V_0 w_i.$$

We may then restate (2) as

$$I_{\text{syn}}(t) = y_2 - y_3. \tag{6}$$

We hence incorporate the effects of all previous spikes, without the need to store individual spike times (Brette et al., 2007). Eq. (6) is possible because Eq. (5) are both linear.

We may now restate (1) as

$$C\frac{dV}{dt} = C\frac{dy_1}{dt} = -gy_1 + y_2 - y_3, \tag{7}$$

and we may restate (7) and (5) in matrix form,

$$\frac{d\mathbf{y}}{dt} = \mathbf{A}\mathbf{y} = \begin{bmatrix} -g/C & 1/C & -1/C \\ 0 & -1/\tau_1 & 0 \\ 0 & & -1/\tau_2 \end{bmatrix} \begin{bmatrix} y_1 \\ y_2 \\ y_3 \end{bmatrix} \tag{8}$$

where $\mathbf{y}$ represents the state vector of the system. The solution of (8) is equivalent to (3),

$$\mathbf{y}(t) = e^{\mathbf{A}t}\mathbf{y}(0),$$

where $e^{\mathbf{A}t}$ is known as the matrix exponential. As the exponential function is defined by its 'series expansion', $e^a = \sum_{i=0}^{\infty} \frac{a^n}{n!}$, so the matrix exponential function is defined by a corresponding series expansion, $e^{\mathbf{A}} = \sum_{i=0}^{\infty} \frac{\mathbf{A}^n}{n!}$, where $\mathbf{A}^n = \mathbf{A} \times \mathbf{A} \ldots \times \mathbf{A}$ (n times) represents matrix multiplication. For a given time step h, Matlab derives $\mathbf{P}(h) = e^{\mathbf{A}h}$ in symbolic form,

```
A=sym('[-h*g/C, h/C, -h/C; 0, -h/tau_1, 0; 0, 0, -h/tau_1]')
matrix_exponential=expm(A);
pretty(matrix_exponential)
```

which finally gives us

$$\mathbf{P}(h) = \begin{bmatrix} e^{-\frac{hg}{C}} & \frac{\tau_1}{C-g\tau_1}(e^{-\frac{hg}{C}} - e^{-\frac{h}{\tau_1}}) & -\frac{\tau_2}{C-g\tau_2}(e^{-\frac{hg}{C}} - e^{-\frac{h}{\tau_2}}) \\ 0 & e^{-\frac{h}{\tau_1}} & 0 \\ 0 & 0 & e^{-\frac{h}{\tau_2}} \end{bmatrix} \mathbf{y}(0)$$

$\mathbf{P}(h)$ is constant, needs to be calculated only once and, in symmetry with (4), computes $\mathbf{y}(nh)$ exactly for any integer n,

$$\mathbf{y}(nh) = \mathbf{P}(h)^n \mathbf{y}(0).$$

Estimation of continuous spike times

Spikes occur between grid points, and are estimated by interpolation. For instance, consider a membrane potential V that has crossed its threshold V_{thr} in the interval $[t, t+h]$. Linear interpolation would estimate the spike time t_{spike} as

$$t_{\text{spike}} \approx t + \frac{V_{\text{thr}} - V(t)}{V(t+h) - V(t)}.$$

Quadratic or cubic interpolation may also be used and may be more precise.

Estimations of spike times introduce a number of technical caveats, involving changes in the timing of the post-synaptic potential and emergence from refractory period. These technical issues are discussed in detail in Morrison et al. (2007), section 4.2.

References

1. Morrison A et al. (2007) Exact subthreshold integration with continuous spike times in discrete-time neural network simulations. *Neural Computation* 19:47-79.

2. Burkitt AN (2006) A review of the integrate-and-fire neuron model: I. Homogeneous synaptic input. *Biological Cybernetics* 95:97-112.

3. Brette R et al. (2007) Simulation of networks of spiking neurons: A review of tools and strategies. *Journal of Computational Neuroscience* 23:349-398.
